# Comparison of the morphogenesis of three genotypes of pea (*Pisum sativum*) grown in pure stands and wheat-based intercrops

**DOI:** 10.1093/aobpla/plu006

**Published:** 2014-02-18

**Authors:** Romain Barillot, Didier Combes, Sylvain Pineau, Pierre Huynh, Abraham J. Escobar-Gutiérrez

**Affiliations:** 1 LUNAM Université, Groupe Ecole Supérieure d'Agriculture, UPSP Légumineuses, Ecophysiologie Végétale, Agroécologie, 55 rue Rabelais, BP 30748, F-49007 Angers Cedex 01, France; 2 Present address: INRA, Centre de Versailles-Grignon, U.M.R. INRA/AgroParisTech Environnement et Grandes Cultures, 78850 Thiverval-Grignon, France; 3 INRA, UR4 P3F, Equipe Ecophysiologie des plantes fourragères, Le Chêne – RD 150, BP 6, F-86600 Lusignan, France

**Keywords:** Morphogenesis, *Pisum sativum*, plant architecture, plasticity, *Triticum aestivum*, wheat–pea intercropping.

## Abstract

Cereal–legume intercrops represent a promising way of combining high productivity and agriculture sustainability. The benefits of cereal–legume mixtures are highly affected by species morphology and functioning, which determine the balance between competition and complementarity for resource acquisition. Studying species morphogenesis, which controls plant architecture, is therefore of major interest. The morphogenesis of cultivated species has been mainly described in mono-specific growing conditions, although morphogenetic plasticity can occur in multi-specific stands. The aim of the present study was therefore to characterize the variability of the morphogenesis of pea plants grown either in pure stands or mixed with wheat. This was achieved through a field experiment that included three pea cultivars with contrasting earliness (hr and HR type) and branching patterns. Results show that most of the assessed parameters of pea morphogenesis (phenology, branching, final number of vegetative organs and their kinetics of appearance) were mainly dependent on the considered genotype, which highlights the importance of the choice of cultivars in intercropping systems. There was however a low variability of pea morphogenesis between sole and mixed stands except for plant height and branching of the long-cycle cultivar. The information provided in the present study at stand and plant scale can be used to build up structural–functional models. These models can contribute to improving the understanding of the functioning of cereal–legume intercrops and also to the definition of plant ideotypes adapted to the growth in intercrops.

## Abstract (precis)

In intercropping systems, plant morphology highly determines the amount of resources captured by each component species. However, morphogenesis of cultivated species has been mainly described in mono-specific growing conditions, although plasticity can occur in multi-specific stands. This paper reports on the variability of the morphogenesis of three pea genotypes grown in pure stands and mixed with wheat. Most morphogenetic parameters of pea were dependent on the genotype. However, there was low variability of pea morphogenesis between sole and mixed stands, except for plant height and branching of the long cycle cultivar.

## Introduction

In order to ensure agriculture sustainability, efforts have been made by researchers and farmers to reduce the use of fertilizers and pesticides. This challenges the maintenance of efficient and profitable agrosystems being able to face demographic growth. Because of their ability to fix atmospheric N_2_, legume species can improve the sustainability of cropping systems by helping to decrease the use of nitrogen fertilizers and favouring the diversification of crop rotations (Crews and Peoples 2004; Duc *et al.* 2010). Also, seeds or forages of legumes are among the richest sources of proteins in crops, with a high nutritional value for animals (Duc *et al.* 2010). However, the potential productivity of legumes has not been reached, mainly because of a strong sensitivity of these species to lodging and foliar diseases (Ney and Carrouée 2005). This is in particular the case for pea (*Pisum sativum*), which is the main source of vegetable proteins in Europe. In this context, the increasing interest in growing cereal–legume intercrops (IC), such as wheat–pea mixtures, represents an alternative for reintroducing legume species in cropping systems. Several studies reported that these mixtures can provide high and stable yields compared with pure mono-specific stands (Ofori and Stern 1987; Jensen 1996; Corre-Hellou *et al.* 2006; Hauggaard-Nielsen *et al.* 2008). Such advantages result from a balance between complementary (e.g. separate root and canopy areas) and competition processes for light, water and nitrogen that occur between intercropped species. These complex interactions depend on the pedo-climatic conditions, agricultural practices and also on the morphology and functioning of the component species (Corre-Hellou *et al.* 2006; Launay *et al.* 2009; Louarn *et al.* 2010; Naudin *et al.* 2010; Barillot 2012).

The latter point is mainly related to the choice of cultivars, which therefore appears as a determinant factor of (i) the proportion of each component species at harvest and (ii) mixture productivity. Cultivars are usually discriminated according to their earliness, sensitivity to diseases or potential yield. However, in the particular case of multi-specific stands, the above-ground architecture of a cultivar, given by its geometry, optical properties and topology of the phytoelements (Godin 2000), should also be taken into account. Indeed, plant architecture defines the plant interface with biotic (e.g. with *Mycosphaerella pinodes*; Béasse *et al.* 2000; Le May *et al.* 2009) and abiotic factors (e.g. light; Ross 1981). In the case of multi-specific stands, the complementarity between the architecture of the mixed species represents a crucial issue as it will determine their respective ability to compete for light that in turn drives the production and allocation of biomass (Varlet-Grancher *et al.* 1993; Sinoquet and Caldwell 1995).

For pea, several genes involved in the development of the above-ground architecture have been identified (for a review see Huyghe 1998). For instance, numerous *ramosus* mutants were described because of their altered branching behaviour (Arumingtyas *et al.* 1992). Plant height can also be altered through mutations made on genes involved in internode growth (Kusnadi *et al.* 1992)*.* Genetic control of the compound leaf shape of pea has also been assessed and appears to be related to the *UNIFOLIATA* gene (Gourlay *et al.* 2000). Precocity of pea cultivars has been shown to be regulated by genes (*Hr* and *Lf*) that control the sensitivity to photoperiod for floral initiation and flowering (Murfet 1973, 1975). These studies have promoted the breeding of several pea cultivars with contrasting architectures that therefore constitute as much as potential combinations for wheat–pea IC. Characterizing the morphogenesis (sequence of developmental and growth processes leading to plant architecture) of these various pea genotypes is therefore of major interest for improving the management of intercropped stands. Several descriptors can be used to characterize pea architecture, the most commonly used being those related to the leaf area and its spatial distribution as this strongly determines a plant's ability to compete for light interception. On a finer scale, both the amount and distribution of foliar area are related to the number and geometry of stems and leaves produced during the initiation of each phytomer by the apex. A phytomer is defined as a basic unit repeated along the stem and including an internode, a node, a leaf and one or several axillary buds (Gray 1849; White 1979). The sharing of resources within multi-specific stands also depends on the respective height reached by the component species (Sinoquet and Caldwell 1995; Schwinning and Weiner 1998; Louarn *et al.* 2010; Barillot *et al.* 2011, 2012). Although the architectural parameters involved in the leaf area and height of plants are key factors of the mixture development, they have been mainly described in mono-specific growing conditions (for a review see Munier-Jolain *et al.* 2005*a*). However, the morphogenesis of plants can be highly plastic when facing environmental variations; hence the question arises as to whether morphogenetic variations can occur between mono- and multi-specific stands.

The aim of the present study was therefore to characterize the variability of pea morphogenesis grown either in pure stands or mixed with wheat. In order to have a large range of plant architectures and morphogenetic responses, a field experiment was performed using three pea cultivars with contrasting growth habits. The growth and phenology of the pea cultivars were measured regularly during their growing cycle. This study provides information at both stand and plant scale in order to identify plant traits of interest that can contribute to the conception of plant ideotypes.

## Methods

### Plant material and growing conditions

A field experiment was carried out in 2010–11 at Brain-sur-l'Authion, western France (47°26′N, 00°26′W) in a clay soil (51 % clay, 26 % silt and 23 % sand). Daily mean air temperature, precipitation and photosynthetically active radiation (PAR) were recorded by a standard automatic agro-meteorological station located close to the field.

Winter wheat (*Triticum aestivum*) cv. Cézanne and three cultivars of winter field pea (*Pisum sativum*), cv. Lucy (hr type), AOPH10 (hr type) and 886/01 (HR type), were sown on 28 November 2010 in sole crops (SC) and IC. Unlike hr types, flowering of the HR cultivar is sensitive to photoperiod. The sowing density of SC was 250 plantsm^−2^ for wheat. Optimal densities of pea cultivars were chosen with respect to their ability for lateral development and the underlying risk of lodging. Sole crops composed of pea cultivars Lucy and AOPH10 were sown at 80 plantsm^−2^ whereas cultivar 886/01 was sown at 40 plantsm^−2^. Intercrops followed a substitutive design where the two species were mixed within the row. Wheat and pea crops in IC were sown at half their respective density in pure stands. From seedbed preparation to harvest, local agronomic recommendations were followed and pest and weed were chemically controlled. Stands of sole wheat were fertilized with 14 g N m^−2^ whereas pea SC and wheat–pea IC were not supplied with external nitrogen.

Statistical analyses described below were thus performed considering two factors: (i) pea genotype with three levels (Lucy, AOPH10 and 886/01) and (ii) cropping system with two levels (SC and IC). A sole crop of wheat was added to those six treatments but was not considered for statistical analyses. These seven treatments (3 SC of pea, 3 IC and 1 SC of wheat) were arranged in experimental units of 1.2 × 10 m^2^ within a randomized complete block design with three replicates.

### Plant sampling and measurement of pea morphogenesis

On the one hand, integrated parameters defined at canopy scale (biomass, height, yield) were measured in each plot. Samplings were carried out on 0.75 m^2^ in the centre of each experimental unit. The above-ground biomass and the maximal height of each SC (wheat and pea) and IC plot were measured during the growing cycle at 645, 1525 growing degree days (GDD) from emergence (base temperature 0 °C) and, lastly, at crop maturity (Table 1). The land equivalent ratio (LER) for grain yields of wheat–pea IC was also estimated according to De Wit and Van den Bergh (1965). Land equivalent ratio is the sum of partial LER values for wheat (LER_wheat_) and pea (LER_Pea_):

$$\hbox{LER}_{{\rm wheat}} = \displaystyle{{Y_{{\rm ICw}} } \over {Y_{{\rm SCw}} }}, \quad \hbox{LER}_{{\rm pea}} = \displaystyle{{Y_{{\rm ICp}} } \over {Y_{{\rm SCp}} }}, \quad \hbox{LER = LER}_{{\rm wheat}} + \hbox{LER}_{{\rm pea}} $$

where *Y*_ICw_ and *Y*_SCw_ are yields of wheat and pea in IC, respectively, and *Y*_SCw_ and *Y*_SCp_ are yields of wheat and pea in SC, respectively. Land equivalent ratio values above 1 indicate a benefit of intercropping over sole cropping.

**Table 1.** Harvest time, expressed in growing degree day (DD) from emergence (base, 0 °C), of pea and wheat grown in SC and in IC.

Species	Genotype	Harvest time in SC (DD)	Harvest time in IC (DD)
Pea	Lucy	1900	2275
Pea	AOPH10	1985	2275
Pea	886/01	2130	2275
Wheat	Cézanne	2275	2275

On the other hand, specific measurements on pea cultivars were made at plant scale. The morphogenesis of five pea plants per plot was characterized for each vegetative axis, i.e. main stems and lateral branches. Only one branch at each nodal position of the main stem was followed up. Branches were denoted according to their topological position, i.e. main stems were denoted as Axis-0, then branches that emerged from node *n* of the main stem were referenced as Axis-*n*. For each axis group, the kinetics of phytomer appearance (unfolding leaf visible to the naked eye) were measured and fitted with Schnute's non-linear model (Schnute 1981) using the least-squares method. The model is written as

$$y = \left[ {y_{\max }^B \displaystyle{{1 - \hbox{e}^{ - A(t )} } \over {1 - \hbox{e}^{ - A({t_{\max } } )} }}} \right]^{1/B} + \, \varepsilon _i $$

where *y* is the number of visible phytomers; estimated parameters are *A* and *B* which implicitly define the shape of the curve; *t*_max_ is the last value of the time (*t*) domain for which the model is fitted, corresponding to the end of the vegetative development of the stem; parameter *y*_max_ is the value of *Y* at *t*_max_ and *ɛ* is the residual. Parameters were optimized using the Levenberg–Marquardt iterative method (Marquardt 1963) with automatic computation of the analytical partial derivatives. The fitting procedure was performed for each axis group of each plant. The first derivatives of Schnute's adjustments were also used in order to estimate the rates of phytomer production of the pea cultivars.

### Statistical analyses

Exploratory data analysis, analysis of variance and non-linear regression techniques were performed with R software (R Development Core Team 2012). Analyses of variance (ANOVAs) were performed following a two-factor linear model such that

$$y_{ijk} = \mu + B_i + G_j + C_k + ({G \times C} )_{\,jk} + \varepsilon _{ijk} $$

where *Y* is any dependent variable, *µ* the mean value of *Y*, *B_i_* the effect of block *i*, *G_j_* the effect of genotype *j*, *C_k_* the effect of cropping system *k* (either SC or IC) and *ɛ* the random error of measurement *ijk*.

Normal distributions of the residuals of ANOVAs as well as those of Schnute's adjustments were tested using the Shapiro–Wilk test. Homoscedasticity was checked by random distribution of the residuals. Tukey's HSD tests were used for mean separation when three or more means were compared.

## Results

**Figure 1.**
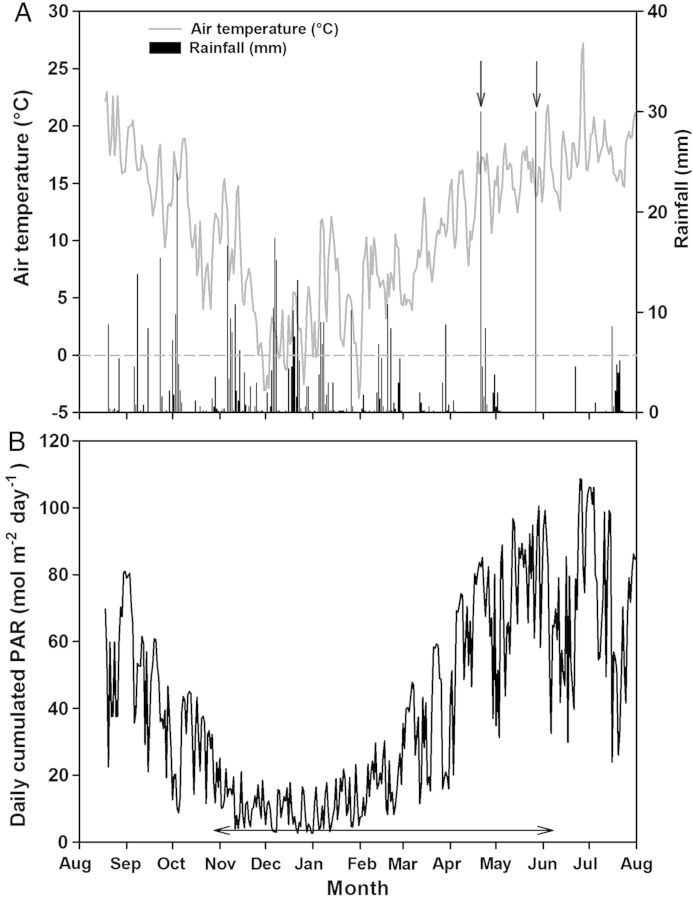
Meteorological conditions during the growing season 2010–11 at Brain-sur-l'Authion, France. Daily mean air temperatures and rainfall are shown in (A). Vertical arrows represent a water supply of 30 mm by irrigation. Daily cumulated PAR is shown in (B). The horizontal arrow represents the growing period of pea and wheat stands.

Environmental conditions during crop growth are summarized in Fig. 1. The daily average air temperature ranged from −4 °C to 27 °C on 31 January and 27 June, respectively (Fig. 1A). Irrigation supplied 30 mm of water on 21 April and 27 May 2011. The daily cumulated PAR (Fig. 1B) ranged from 2.70 to 108.70 mol m^−2^ on 31 December 2010 and 25 June 2011, respectively.

### Growth of SC and IC stands

**Figure 2.**
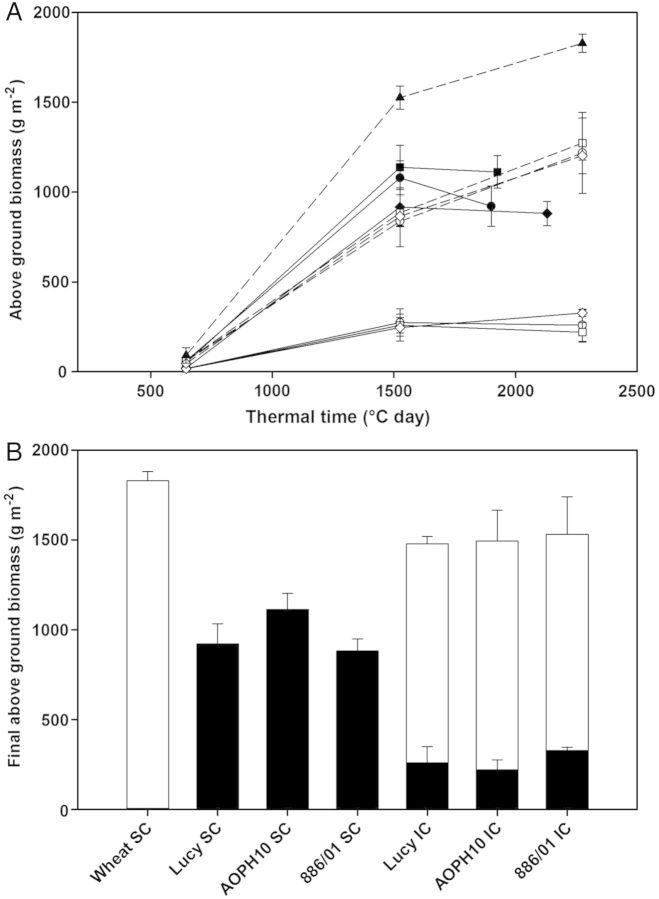
(A) Accumulation of above-ground biomass as a function of thermal time from emergence (base temperature = 0 °C). Sole crops are shown in closed symbols and IC in open symbols. Pea cultivars are in solid lines: Lucy is denoted by circles (filled, open), AOPH10 by squares (filled, open) and 886/01 by diamonds (filled, open). Wheat is in dotted lines with triangles (filled) in SC, and with the corresponding pea cultivar symbol in IC (open circles with ‘Lucy’, open squares with ‘AOPH10’ and open diamonds with ‘886/01’). (B) Contribution of wheat (white bars) and pea (black bars) in the final biomass reached by SC and IC stands.

The results described in this section are derived from measurements at the whole stand scale. First, the biomass accumulation of each crop is shown in Fig. 2. The above-ground biomass of the two species increased from 645 to 1525 GDD and then slowed down until maturity (Fig. 2A). Wheat SC showed the highest amount of biomass during the growing cycle and finally reached 1830 g m^−2^. The final above-ground biomass accumulated by the three pea cultivars in SC ranged from 880 to 1275 g m^−2^. On average, ‘886/01’ exhibited the lowest biomass in SC throughout the growing cycle and finally reached 880 ± 67 g m^−2^. This lower biomass has to be related to its sowing density, which was 50 % of the other cultivars (see the Methods section). Intercropped wheat (IC stands) accumulated on average 1230 g m^−2^ of biomass of all IC stands taken together. Pea grown in IC stands produced 220–325 g m^−2^ of biomass at maturity. At this stage of development, the final biomass reached by IC stands was 1500 g m^−2^ averaged across pea cultivars (Fig. 2B). Wheat in IC contributed to the main part of the mixture biomass (on average 3.75 times the biomass of pea). Although cultivar 886/01 exhibited the lowest biomass in SC stands, this cultivar produced more biomass when intercropped with wheat compared with ‘Lucy’ and ‘AOPH10’, despite being sown at half density.

Harvest of IC stands (Table 1) was performed when wheat reached maturity (2275 GDD). For pea grown in SC, harvest was made earlier, i.e. between 1900 and 2130 GDD depending on the cultivar. Cultivars Lucy and AOPH10 (hr types) reached their maturity earlier than ‘886/01’. Indeed, cultivar 886/01 (HR type) needs a longer photoperiod to reach flowering and was therefore harvested later than hr cultivars. As a result, the maturity of cultivar 886/01 and that of wheat were reached in a similar period (2130 and 2275 GDD, respectively).

**Figure 3.**
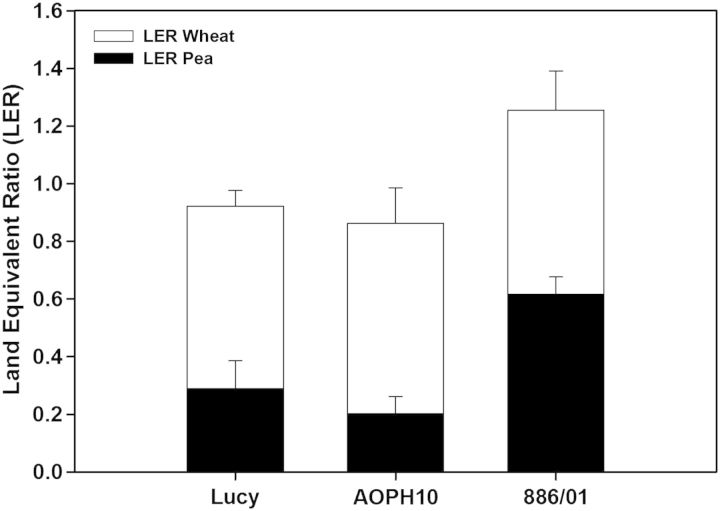
Land equivalent ratio of each wheat–pea mixture. Land equivalent ratio values >1 indicate a benefit of intercropping over sole cropping. *N.B.:* unlike pure stands of pea and IC, wheat SC were fertilized with external nitrogen.

Land equivalent ratios for grain yield (LERs) were estimated for IC (Fig. 3). Although wheat SC stands were fertilized with nitrogen and IC stands were not, the partial LER of wheat was systematically higher than 0.5 (0.65 on average) whatever the companion pea cultivar. Thus, wheat yields in IC were higher than those in SC when normalized by the sowing density (wheat density in IC was half that of SC but yields were reduced by <35 %). Land equivalent ratios of IC stands composed of cultivars Lucy and AOPH10 were slightly <1, meaning that the cumulative yield of wheat and pea in IC stands was lower than the sum of each SC yield. Analysis of partial LERs of ‘Lucy’ and ‘AOPH10’ (0.3 and 0.2, respectively) revealed that their yield markedly decreased in IC compared with pure stands. In contrast, the yield of IC based on cultivar 886/01 was 25 % higher than the cumulative yield of wheat and ‘886/01’ in SC. In these IC stands, the yield of ‘886/01’ was strongly increased compared with SC conditions (LER_886/01_ = 0.62 on average). Interestingly, the ratio of the final above-ground biomass of ‘886/01’ in IC to that of ‘886/01’ in SC (0.37, Fig. 2B) was smaller than the grain yield ratio (LER_866/01_). This means that the harvest index (ratio grain yield/above-ground biomass) was particularly high in intercropped ‘886/01’.

**Figure 4.**
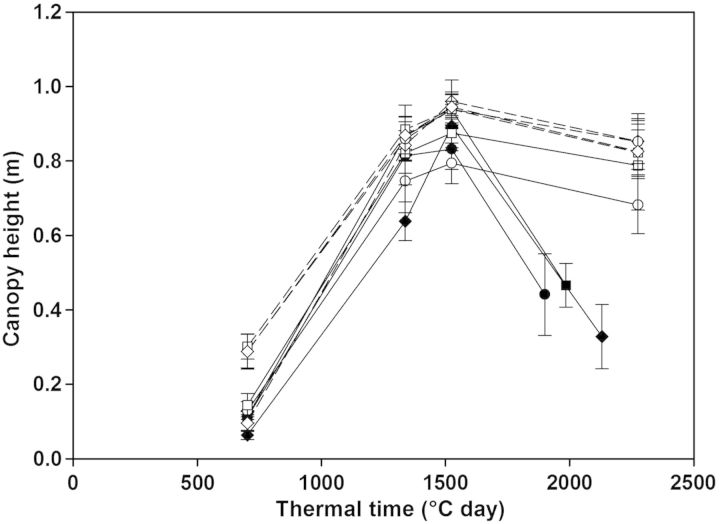
Observed height of canopies during the growing cycle. Sole crops are shown in closed symbols and IC in open symbols. Pea cultivars are in solid lines: Lucy is denoted by circles (filled, open), AOPH10 by squares (filled, open) and 886/01 by diamonds (filled, open). Wheat is in dotted lines with triangles (filled) in SC, and with the corresponding pea cultivar symbol in IC (open circles with ‘Lucy’, open squares with ‘AOPH10’ and open diamonds with ‘886/01’).

Wheat plants grown in SC and IC stands exhibited similar height and reached a maximum of 0.95 m (Fig. 4). Wheat grown in IC was taller than pea from the early stages of development and whatever the pea cultivar. The height reached by the pea cultivars was variable, depending on the cropping system (SC or IC stand). The canopy height of sole pea crops reached a maximum of 0.83–0.94 m for ‘Lucy’ and ‘AOPH10’, respectively. The height of sole pea finally decreased dramatically at the end of the growing cycle as a result of plant lodging. However, the height of pea cultivars grown in IC stands remained over 0.70 m, meaning that pea plants grown in IC were staked by wheat stems thus preventing pea lodging. The species height ratio (pea/wheat) in IC is shown in Table 2. The height ratio ranged from 0.33 to 0.48 at 700 GDD, meaning that pea cultivars were strongly dominated in the first stages of development, especially for ‘886/01’. From 1330 GDD on, the Lucy and AOPH10 cultivars were slightly shorter than wheat, whereas ‘886/01’ reached the height of wheat.

**Table 2.** Height ratio of wheat–pea mixtures according to the growing degree day (GDD).

Genotype	Height ratio (pea/wheat)			
	700 GDD	1330 GDD	1525 GDD	2275 GDD
Lucy	0.45	0.87	0.85	0.80
AOPH10	0.48	0.93	0.93	0.96
886/01	0.33	0.97	1.02	1.03

### Morphogenesis of pea cultivars

In a second step, measurements were made at plant scale in order to compare the morphogenesis of pea cultivars grown in pure stands with those intercropped with wheat.

#### Lateral branching of pea plants

**Figure 5.**
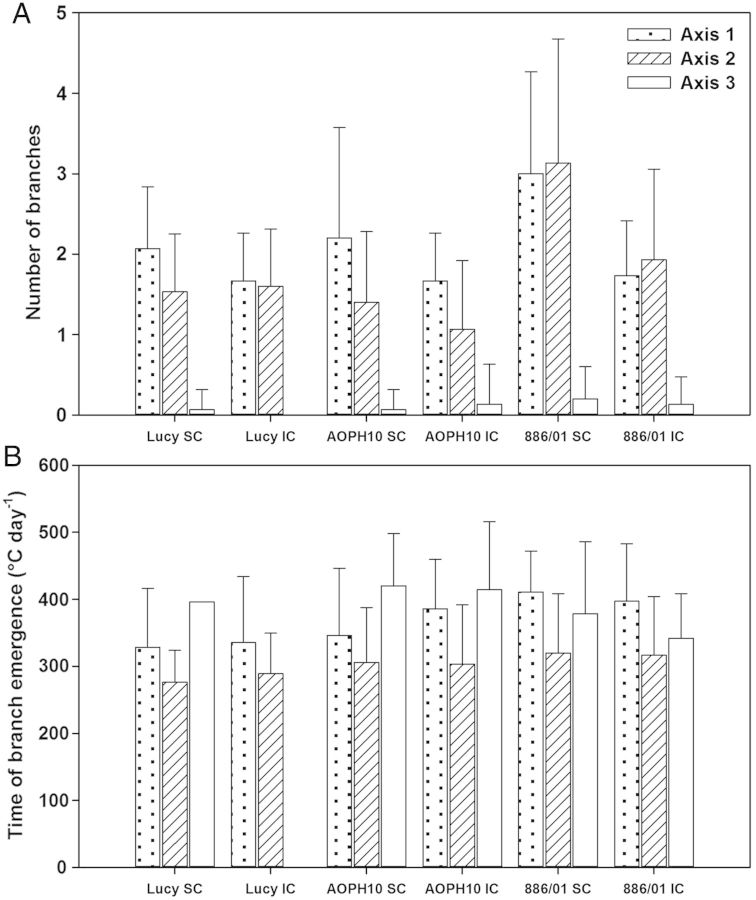
Lateral branching of pea cultivars (*n* = 15 plants for each condition). (A) Number of lateral branches developed by the pea cultivars Lucy, AOPH10 and 886/01 grown in SC and in IC. (B) Time of branching expressed in thermal time from crop emergence. Branches were distinguished according to their nodal position on the main stem. Axis-1: branches emerged at the first node; Axis-2: second node; and Axis-3: third node.

The total number of branches (including non-flowering ones) produced by individual pea plants is shown in Fig. 5A according to their nodal position on main stems. Most lateral branches emerged from the first and second phytomer of the main stems (Axis-1 and -2) whatever the cropping system (SC and IC) and pea cultivar. Very few branches were produced on the third phytomer of the main stems (only nine branches, all cultivars and cropping systems taken together). Cultivars Lucy and AOPH10 grown in SC developed on average 3–4 branches per plant whereas cultivar 886/01 in SC was the most branching with about 6 branches per plant on average. Nevertheless, a significant effect of the genotype on the number of branches was only found for Axis-2 (ANOVA *F*_2,82_ = 13.93, *P* < 0.001). Indeed, cultivar 886/01 produced significantly more branches of type Axis-2 than Lucy and AOPH10 cultivars did (HSD *P*< 0.001). The ability of the 886/01 cultivar to develop numerous lateral branches appeared to compensate for its lower sowing density as shown by its biomass accumulation, which was similar to those of cultivars Lucy and AOPH10 (Fig. 2). Significant effects of the cropping system on the number of Axis-1 (*F*_1,82_ = 14.33, *P*< 0.001) and Axis-2 (*F*_1,82_ = 5.48, *P*< 0.05) were also found. Indeed, pea cultivars grown in IC tended to develop fewer branches than in SC. The most drastic decrease was observed for cultivar 886/01, which produced 40 % less branches in IC (HSD *P* < 0.01 and <0.05 for Axis-1 and -2, respectively). However, for ‘Lucy’ (−11 %) and ‘AOPH10’ (−22 %) intercropped with wheat, it was not possible to detect any significant differences in the number of branches between the two cropping systems, probably because of the high variability observed.

Lateral branches emerged between 275 and 420 GDD, all cultivars and cropping systems taken together (Fig. 5B). Although they were initiated later, branches that developed on the second phytomer of the main stem (Axis-2) appeared 70 GDD earlier than those located on the first node (Axis-1). Indeed, in most cases, the first phytomer (carrying the first vestigial leaf) is located a few millimetres under the ground. This may mechanically delay the emergence of branches, depending for instance on the sowing depth or soil structure, which also affect the quantity of light perceived by the axillary bud (Jeudy and Munier-Jolain 2005). Statistical analyses showed that there was a significant effect of the genotype on the time of branch emergence (*F*_2,87_ = 5.09, *P*< 0.01; *F*_2,166_ = 3.20, *P*< 0.05 for Axis-1 and -2, respectively). Indeed, branches developed on cultivar Lucy emerged significantly earlier than those of ‘886/01’ (HSD *P* < 0.01 and <0.05 for Axis-1 and -2, respectively), while ‘AOPH10’ had an intermediary behaviour. There were however no significant differences in the time of branch emergence between SC and IC stands, meaning that this parameter of pea morphogenesis was mainly dependent on the genotype.

**Figure 6.**
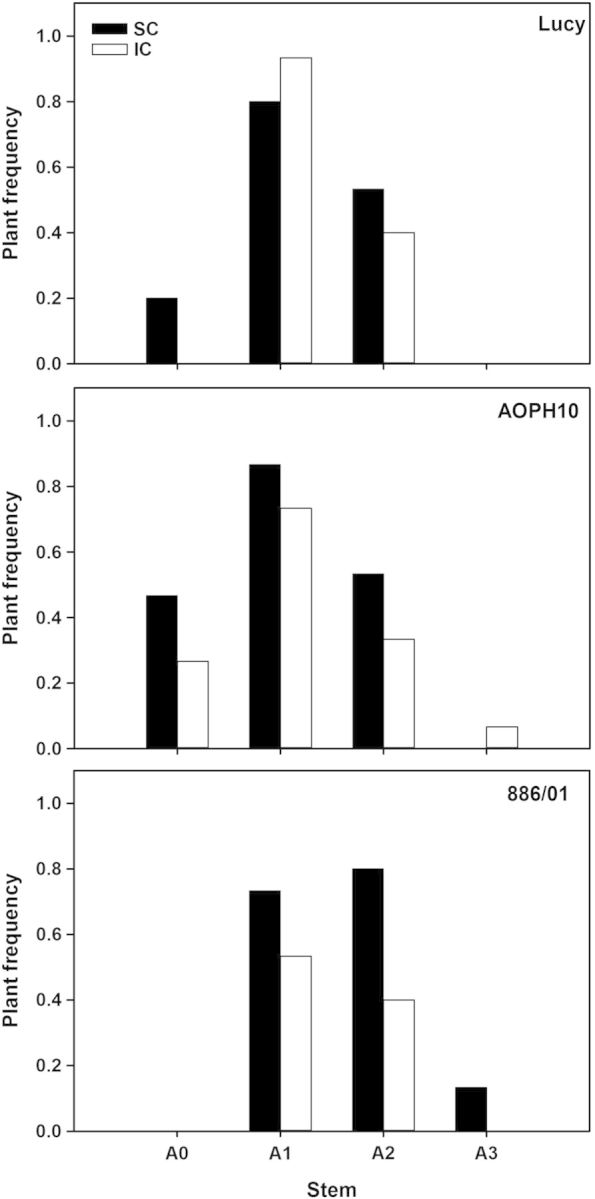
Frequency of pea plants grown in SC and in IC whose measured branches have reached flowering. Branches were distinguished according to their nodal position on the main stem. Axis-1: branches emerged at the first node; Axis-2: second node; and Axis-3: third node (*n* = 15 plants for each condition, only one single branch was considered at each nodal position).

As shown above, pea genotypes produced several branches during the first 500 GDD. This led to complex plant architectures with a high potential number of stems per plant. For the sake of clarity, we only consider hereafter one branch at each node of the main stems. As a result, plant architectures were simplified to a main stem potentially bearing one branch on its first three nodes (see Fig. 5A). However, only a part of these stems actually grew and completed their development up to flowering. As shown in Fig. 6, very few main stems of pea flowered. This was in particular the case of cultivars 886/01 (0 % of flowering main stems) and Lucy (0–20 % of flowering main stems for IC and SC, respectively). Flowering of main stems was significantly dependent on the cultivar (*F*_2,10_ = 6.83, *P*< 0.05). Indeed, the proportion of ‘AOPH10’ plants that had flowering main stems (25–45 % in IC and SC, respectively) was statistically higher than that of cultivar 886/01 (HSD *P* < 0.05). Nevertheless, most flowering stems of the three pea cultivars were Axis-1 and -2 branches. For cultivars Lucy and AOPH10, there were 85 % more of Axis-1 branches that carried on growing until flowering compared with Axis-2 (regardless of the cropping system). In contrast, the proportion of flowering Axis-1 of cultivar 886/01 was similar to Axis-2. Pea plants grown in SC and those intercropped with wheat exhibited similar proportions of flowering stems.

#### Rate of phytomer appearance and final number of phytomers

**Figure 7.**
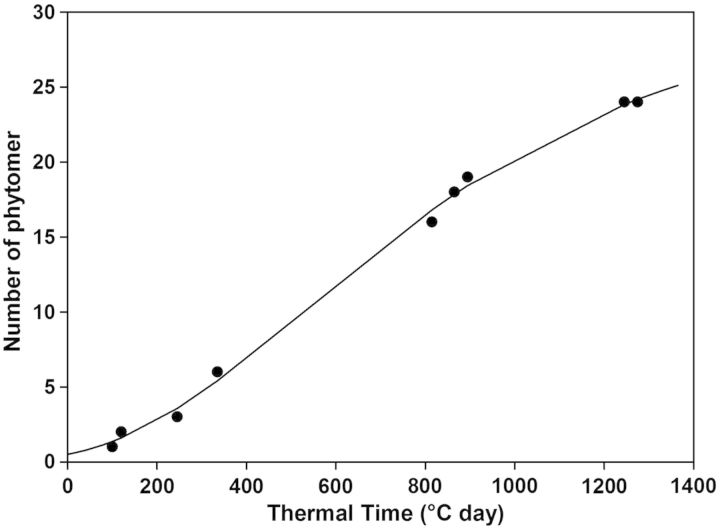
Typical kinetics of phytomer appearance on a vegetative stem of the pea cultivars. Observed values are in closed symbols. Non-linear adjustment (solid line) was performed using Schnute's equation.

Phytomer production by stem apices followed sigmoid-type dynamics as illustrated in Fig. 7. These dynamics were fitted with Schnute's function. In order to reduce the variability on Schnute's parameters (Table 3), stems that stop growing before flowering were not taken into account in the fitting procedure. The first three parameters of Schnute's function, *A*, *B* and *t*_max_, respectively, ranged from 0.36 to 4.30 × 10^−3^, 0.13 to 0.56 and 1164 to 1476 GDD averaged across cultivars and cropping systems (SC and IC). The shape parameters *A* and *B* of Axis-2 branches appeared to be significantly dependent on pea cultivar (*F*_2,33_ = 6.43, *P* < 0.01; *F*_2,33_ = 12.23, *P*< 0.001 for parameters *A* and *B*, respectively). Indeed, parameters *A* and *B* of Axis-2 branches produced by cultivar 886/01 were statistically higher than those of ‘Lucy’ and ‘AOPH10’ whatever the considered cropping system (HSD *P* *<*0.05 and <0.01 for *A* and *B*, respectively). We did not find any significant effect of the cropping system on parameters *A* and *B*. The duration of phytomer production (parameter *t*_max_, expressed from stem emergence) appeared to be similar among the different cultivars and cropping systems. Supplementary statistical analyses were also performed in order to compare the kinetics of phytomer production of the different stems. For cultivar Lucy, the kinetics of phytomer production were statistically different between main stems and branches (HSD *P* *<*0.05 and <0.01 for *A* and *B*, respectively). These analyses showed that parameter *B* was significantly different between Axis-1 and -2 branches of cultivar 886/01 (HSD *P* < 0.05).

**Table 3.** Parameters (*A* × 10^−3^, *B* and *t*_max_) of Schnute's adjustments made on the kinetics of phytomer appearance for each pea cultivar (Lucy, AOPH10, 866/01) grown in SC or in IC. Schnute's adjustments were performed for each flowering stem (0: main stem; 1: branch developed on the first node of the main stem; 2: second node). Indicated values are the mean ± SD (*n* = 15 plants for each cultivar and cropping system).

Axis	Parameter	Stand					
		Lucy SC	Lucy IC	AOPH10 SC	AOPH10 IC	886/01 SC	886/01 IC
0	*A* (×10^−3^)	0.36 ± 0.18	–	2.50 ± 1.54	0.82 ± 1.16	–	–
	*B*	0.56 ± 0.04	–	0.27 ± 0.10	0.45 ± 0.17	–	–
	*t*_max_	1415 ± 148	–	1378 ± 204	1257 ± 82	–	–
1	*A* (×10^−3^)	2.36 ± 1.49	2.80 ± 0.99	4.30 ± 4.74	2.48 ± 2.18	1.88 ± 0.95	2.01 ± 1.89
	*B*	0.19 ± 0.12	0.13 ± 0.11	0.18 ± 0.22	0.24 ± 0.21	0.16 ± 0.13	0.25 ± 0.15
	*t*_max_	1294 ± 190	1288 ± 83	1164 ± 336	1288 ± 234	1279 ± 135	1336 ± 199
2	*A* (×10^−3^)	2.36 ± 0.86	2.63 ± 0.53	2.21 ± 1.38	2.49 ± 0.071	1.86 ± 1.46	2.18 ± 1.27
	*B*	0.18 ± 0.09	0.14 ± 0.04	0.19 ± 0.09	0.17 ± 0.08	0.34 ± 0.08	0.25 ± 0.11
	*t*_max_	1362 ± 111	1352 ± 22	1292 ± 224	1378 ± 50	1476 ± 52	1365 ± 242

The maximum rate of phytomer production (*V*_max_) and time at which it was reached $({t_{V_{\max}}}\!\,)$ were estimated by computing the first derivative of Schnute's adjustments (Table 4). The maximum rate of phytomer production ranged from 0.023 for ‘Lucy’ SC to 0.056 phytomer degree-day^−1^ for ‘AOPH10’ SC, which means that at maximum activity one phytomer appeared each at 20–45 GDD. Parameter *V*_max_ of each axis group was found to be dependent neither on pea cultivar nor on cropping system. The time of maximum rate of phytomer appearance $({t_{V_{\max } } }\!\, )$ was reached between about 640 for ‘AOPH10’ SC and 1285 GDD for ‘Lucy’ SC. Parameter $t_{V_{\max } } $ of flowering main stems was significantly higher for ‘Lucy’ than for cultivar AOPH10 (HSD *P* < 0.05). Pea cultivars were also statistically different for parameter $t_{V_{\max } } $ of Axis-1 and -2 branches (*F*_2,53_ = 5.84, *P*< 0.01; *F*_2,32_ = 37.43, *P* < 0.001, respectively). Compared with ‘Lucy’, the maximal rate of phytomer appearance of cultivar 886/01 was indeed reached significantly later for Axis-1 and -2 (HSD *P* < 0.01 and <0.001, respectively). Parameter $t_{V_{\max } } $ of cultivar 886/01 appeared to be significantly higher than that of ‘AOPH10’ but only for Axis-2 branches (HSD *P* < 0.001). For this axis group, $t_{V_{\max } } $ was also found to be higher in SC (1020 GDD) than in IC (845 GDD; HSD *P*< 0.05).

**Table 4.** Rate of phytomer production of each pea cultivar (Lucy, AOPH10, 866/01) grown in SC or in IC. Maximum rate of phytomer production (*V*_max_, phytomer degree-day^−1^) and time at which it was reached $({t_{V_{\rm max}}\!, \hbox{DD}} )$ are shown (mean ± SD). Computations were carried out for each flowered stem (0: main stem; 1: branch developed on the first node of the main stem; 2: second node) (*n* = 15 plants for each cultivar and cropping system).

Axis	Parameter	Stand					
		Lucy SC	Lucy IC	AOPH10 SC	AOPH10 IC	886/01 SC	886/01 IC
0	*V*_max_	0.023 ± 0.002	–	0.024 ± 0.002	0.033 ± 0.006	–	–
	$t_{V_{\max } } $	1284 ± 171.3	–	641 ± 148	1004 ± 279	–	–
1	*V*_max_	0.026 ± 0.005	0.030 ± 0.004	0.056 ± 0.072	0.026 ± 0.006	0.050 ± 0.020	0.030 ± 0.004
	$t_{V_{\max } } $	965 ± 225	836 ± 125	1035 ± 247	950 ± 259	1141 ± 170	1121 ± 224
2	*V*_max_	0.026 ± 0.004	0.030 ± 0.002	0.034 ± 0.013	0.027 ± 0.003	0.030 ± 0.001	0.030 ± 0.001
	$t_{V_{\max } } $	811 ± 69	761 ± 59	929 ± 100	753 ± 47	1226 ± 207	1025 ± 93

**Figure 8.**
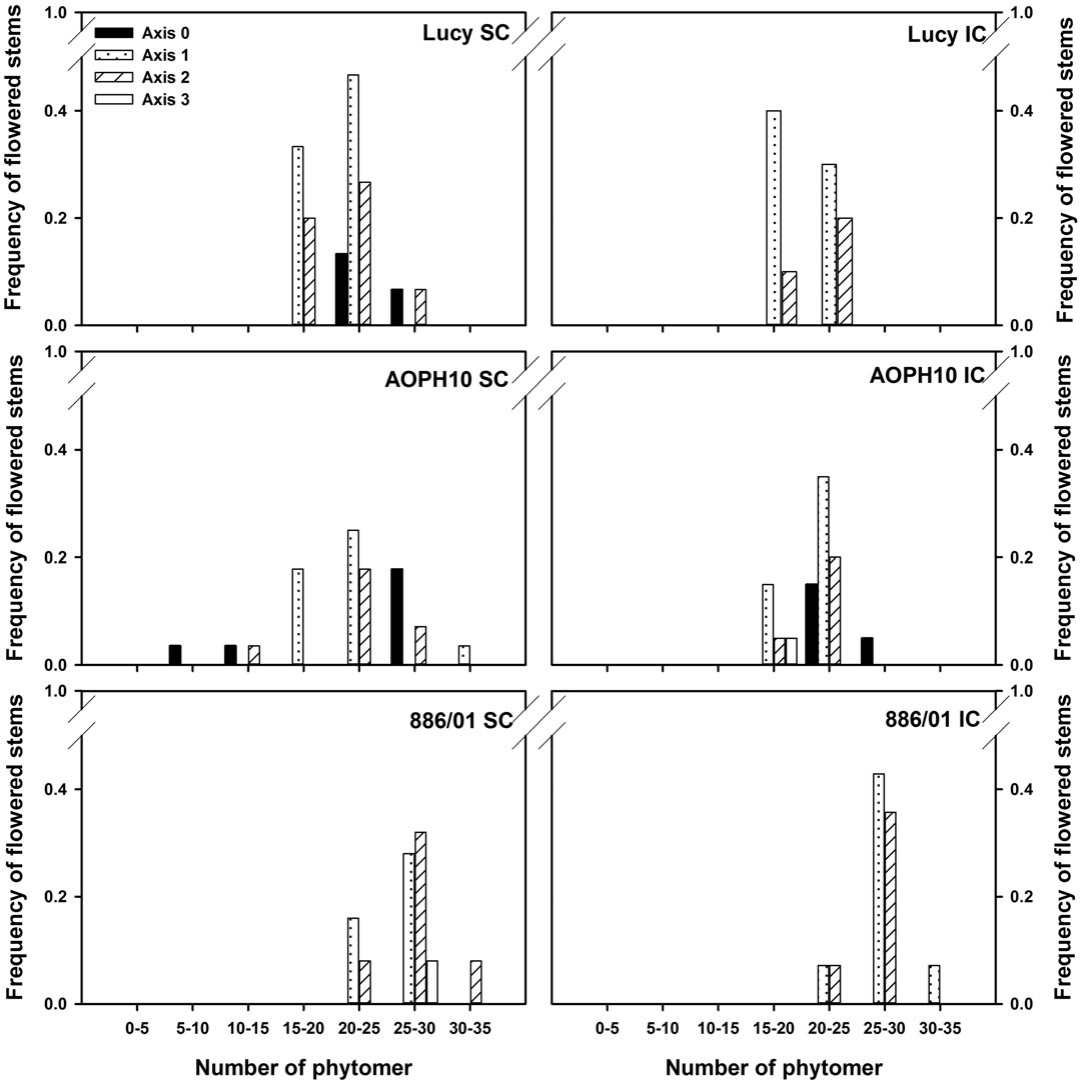
Frequency of flowering stems according to the final number of phytomers. Measurements were made on three pea cultivars: Lucy, AOPH10 and 886/01 grown in SC and in IC with wheat. Results are shown for stems that reached flowering. Main stems are denoted as Axis-0 and branches were distinguished according to their nodal position on the main stem. Axis-1: branches developed at the first node; Axis-2: second node; and Axis-3: third node (*n* = 15 plants for each condition).

The last parameter of Schnute's adjustments to be analysed is the final number of phytomers (*y*_max_). The number of phytomers produced on flowering stems ranged from 9 to 32 averaged across pea cultivars and cropping (Fig. 8). A marginal proportion of flowering stems exhibited <15 phytomers (1 % for ‘AOPH10’, including main stems and Axis-2 branches). Stems with >15 phytomers were mainly branches developed on the first and second phytomer of main stems. This behaviour was however different in the case of cultivar AOPH10, for which ∼20 % of flowering stems were main stems with >20 phytomers. Statistical analyses also showed that the final number of phytomers measured on Axis-1 and -2 branches was dependent on the cultivar (*F*_2,60_ = 58.24, *P*< 0.001; *F*_2,37_ = 25.66, *P* < 0.001 for Axis-1 and -2, respectively). Indeed, cultivar 886/01 produced significantly more phytomers than ‘Lucy’ and ‘AOPH10’ did (HSD *P* < 0.001 for both Axis-1 and -2 branches). The final number of phytomers was not statistically different for pea plants grown in pure stands or intercropped with wheat.

#### Flowering

**Figure 9.**
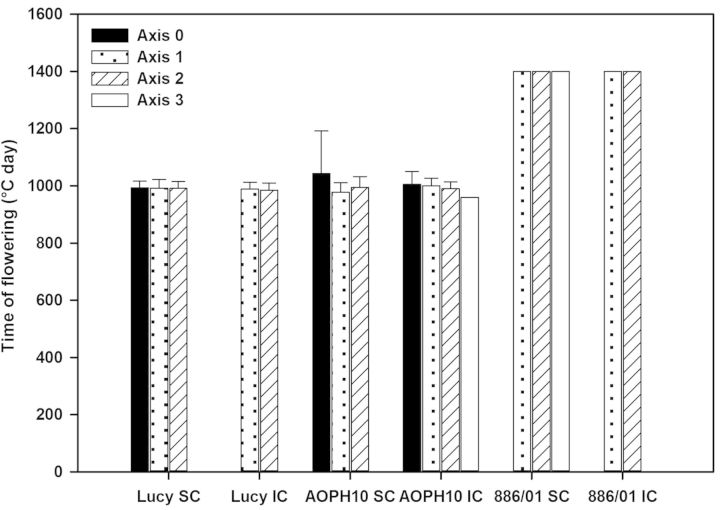
Time of flowering of pea cultivars Lucy, AOPH10 and 886/01 grown in SC or in IC. Main stems are denoted as Axis-0 and branches were distinguished according to their nodal position on the main stem. Axis-1: branches developed at the first node; Axis-2: second node; and Axis-3: third node (*n* = 15 plants for each condition).

**Figure 10.**
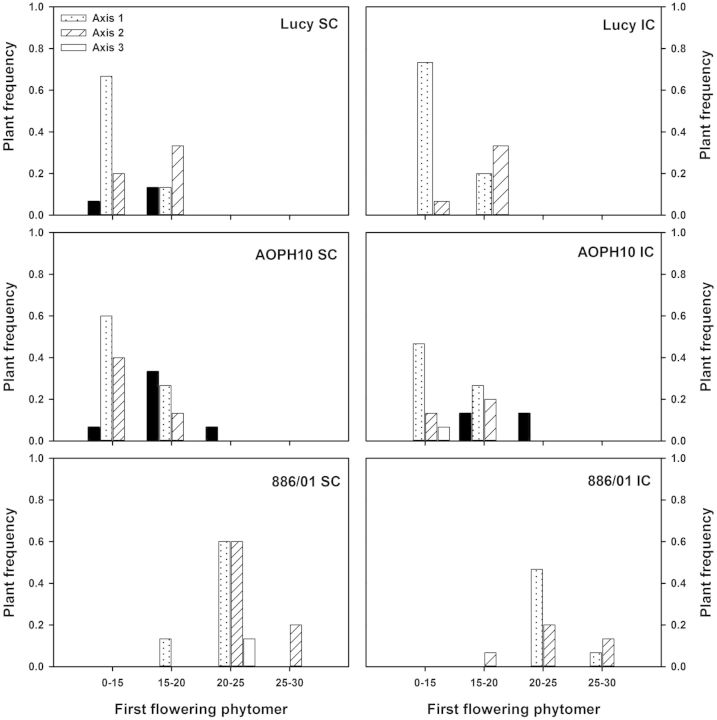
Plant frequency according to the first flowering phytomer of stems. Results are shown for the pea cultivars Lucy, AOPH10 and 886/01 grown in SC or in IC (*n* = 15 plants for each condition). Main stems are denoted as Axis-0 and branches were distinguished according to their nodal position on the main stem. Axis-1: branches developed at the first node; Axis-2: second node; and Axis-3: third node.

The reproductive development of pea cultivars was characterized by the time of flowering of each stem, as well as the nodal position of the first flower. Flowering stage was reached between 950 and 1400 GDD for AOPH10 IC and 886/01, respectively (Fig. 9). For a given cultivar and cropping system, flowering was synchronized between main stems and branches. However, flowering of Axis-1 and -2 branches was significantly different among the pea cultivars (*F*_2,61_ = 1717.29, *P* < 0.001; *F*_2,37_ = 1452.78, *P* < 0.001 for Axis-1 and -2, respectively). Indeed, the flowering stage of cultivar 886/01 was reached significantly later (1400 GDD) than for ‘Lucy’ and ‘AOPH10’ (HSD *P* < 0.001 for both cultivars). The first flowering phytomer (Fig. 10) was located between the ninth and the 28th phytomer averaged across pea cultivars and cropping systems. First flowers of cultivar 886/01 (Axis-1 and -2 branches) were observed at higher phytomer positions (24th phytomer on average) than those measured for ‘Lucy’ and ‘AOPH10’ (HSD *P* < 0.001 for both axis group and cultivars). As observed for the time of flowering, the nodal position of the first flower was similar between pea plants grown in pure stands and those mixed with wheat whatever the cultivars.

## Discussion

The aim of the present study was to address the question of the morphological responses of pea to the competition when intercropped with wheat. To this end, a field experiment was conducted on three pea cultivars that were characterized at both stand and plant scale when grown in pure stands and mixed with wheat.

Although the present study was conducted throughout 1 year only, our results on crop biomass, species height and maturity were consistent with previous studies performed on different cultivars grown under contrasting pedo-climatic conditions (e.g. Corre-Hellou *et al.* 2009; Naudin *et al.* 2010). The results presented in this study can therefore be assumed to be representative of the conditions commonly encountered in wheat–pea mixtures. These results were obtained from unfertilized mixtures as usually performed in cereal–legume intercropping systems. Furthermore, some authors (Jensen 1996; Corre-Hellou *et al.* 2006) found that the contribution of the component species to the biomass of the mixture was dependent on the available nitrogen, an increase of which enhances the growth of the cereal species. The level and timing of nitrogen fertilization (Naudin 2009; Naudin *et al.* 2010) therefore constitute a key factor enabling one to manage the hierarchy between the mixed species. The present study also shows that the three pea cultivars exhibited a similar level of biomass, especially when grown in mixtures. This suggests that despite the genotypic differences and contrasting initial sowing densities, the three pea cultivars had a similar overall development even when they were in competition with wheat. However, this does not necessarily mean that the morphological processes of the pea cultivars had similar responses to the competition with wheat, but their integration at the stand scale leads to an equivalent growth in biomass.

Our results also show that pea was strongly affected by lodging when grown in SC. Corre-Hellou *et al.* (2011) reported that the high sensitivity of pea to lodging caused significant yield losses as well as an enhanced growth of weeds. Pea lodging was however strongly decreased in mixed stands, pea branches being stacked by wheat. Intercropping cereals and legumes is therefore a promising way of both reintroducing legume species within agrosystems and solving the problems encountered in pure stands of legumes. Moreover, the height reached by each species in the canopy is an important feature of the stand which determines, but also emerges from, the competition processes occurring between plants. Component species height ratio has been widely shown to affect light sharing in a mixture (Sinoquet and Caldwell 1995; Louarn *et al.* 2010; Barillot *et al.* 2011, 2012) and is therefore a strong component of the inter-specific competition occurring within the mixture. The results described in the present study illustrate that the species height ratio is not constant throughout the growing cycle; pea cultivars were much shorter than wheat until 700 GDD and then reached a similar height. Differences among pea cultivars were also observed but were not constant over time. Although ‘886/01’ was the shorter one in the early stages of development, this cultivar finally reached the same height as that of wheat afterwards. Therefore, it seems that the competition which occurs among the intercropped species cannot be assessed by punctual measurements of the species height ratio (in particular during the early stages of development). This is consistent with the findings reported in a previous study (Barillot *et al.* 2012) where a virtual plant approach was used to demonstrate that the ability of plants to intercept light was mainly determined by the architectural parameters involved in (i) the LAI (number of branches and phytomers, leaf area) during the early stages and (ii) plant height (internode length, number of phytomers) once canopy closure was established.

The time lag between the physiological maturity of wheat and pea is a well-known issue of these mixtures. The choice of harvest timing is indeed complicated by the fact that pea generally reaches its maturity earlier than wheat. Nevertheless, physiological maturity of pea varies among the cultivars according to their earliness, which is assumed to be mainly driven by the sensitivity of flowering to the photoperiod that involves the *Hr* gene (Murfet 1973). The maturity of the HR cultivar (886/01) was therefore almost synchronized with that of wheat, whereas the hr cultivars had to be harvested earlier. Gaps of maturity, as encountered with hr cultivars, represent a strong practical constraint at harvest (Louarn *et al.* 2010); HR pea cultivars therefore appeared to be well suited to intercropping with wheat.

In order to deepen the analysis of the variability of pea morphogenesis in response to intercropping, a comparison was performed at plant scale with particular attention to pea branching, flowering, final number of phytomers and their kinetics of appearance. Branching has been shown to be dependent on several factors such as genotype, hormonal balance, environmental factors, e.g. low temperatures (Jeudy and Munier-Jolain 2005), and also plant density (Spies *et al.* 2010). In the present study, contrasting abilities for branching were indeed found between the genotypes [Lucy–AOPH10] and 886/01. Cultivar 886/01 was the most branching cultivar, which balanced its lower sowing density (50 % less than ‘Lucy’ and ‘AOPH10’). Moreover, the number of branches tended to decrease in IC compared with pure stands, in particular for cultivar 886/01. Some authors like Casal *et al.* (1986), Ballaré and Casal (2000) or Evers *et al.* (2011) showed that branching of several species is affected by the quantity (PAR) and quality (red/far-red ratio) of light perceived by the axillary buds. In the present study, we can therefore hypothesize that quantity of light and/or its quality were quite similar between the respective pure stands and IC of cultivars Lucy and AOPH10. This would mean that the replacement of a ‘Lucy’ or an ‘AOPH10’ plant by a wheat one leads to similar variations of light microclimate. This could be the result of small differences in the architectural patterns of the two species in terms of leaf area, height, geometry and/or optical properties. As cultivar 886/01 has a late development (HR type), we can also hypothesize that when branching started, wheat plants were more developed than neighbour ‘886/01’ pea plants would have been in a pure stand. This could cause variations of the microclimate perceived by axillary buds, leading to an inhibition of branching.

The kinetics of phytomer appearance were assessed for main stems and a randomly selected branch at each node by using non-linear fittings. Our analysis showed that there were few statistical differences between the parameters belonging to the different genotypes and cropping systems. It was only found that (i) Axis-2 branches of cultivar 886/01 had kinetics different from those of ‘Lucy’ and ‘AOPH10’ and (ii) the maximum rate of phytomer appearance of ‘886/01’ was reached later compared with the other cultivars. These results mean that the kinetics of phytomer production of different stems can be analysed/modelled by using similar Schnute's functions, at least for Lucy and AOPH10 cultivars whether they were grown in sole stands or mixed with wheat. Turc and Lecoeur (1997) also reported similar rates of leaf primordium initiation and emergence for contrasting plant growth rates, cultivars and sowing densities in spring pea. One drawback of using Schnute's function lies in the fact that some of the parameters, especially *A* and *B*, cannot be directly related to a biological meaning. It would be tempting to use linear regressions because of the reduced number of parameters and easy interpretation. However, phytomer production is not intrinsically constant and is actually characterized by a maximum rate (which can be estimated by the derivative of Schnute's functions) and a time at which development stops. These aspects cannot be handled by linear models. Supplementary statistical analyses (data not shown) showed that the residual sum of squares was significantly higher for linear regressions than that obtained with Schnute's function (HSD *P* < 0.001). These tests also indicated that the residuals of most linear regressions were not normally distributed and have means differing from zero. Nevertheless, the estimated parameters derived from Schnute's adjustments were highly variable. This variability is related to pea branching which is rather complex, particularly in the case of winter-sown cultivars. Winter conditions often cause frost damage, which induces the cessation of the development of the main stems and the initiation of numerous branches at different times (Fig. 5A and B). The result is a high variability in the characteristics of branches.

In the present study, a significant difference was observed among the pea genotypes for the final number of phytomers reached on stems. Indeed, ‘886/01’ (HR type) was found to produce more phytomers than the other cultivars. Similar results were also reported for this particular cultivar but grown in controlled conditions and individual pots (Barillot *et al.* 2012). In contrast, the number of initiated phytomers was similar whether pea plants were grown in pure stands or mixed with wheat, whatever the genotype. Moreover, our results show that the canopy of the three pea cultivars was mainly composed of branches as main stems had stopped growing with few phytomers. As reported by Jeudy and Munier-Jolain (2005), the development of branches is increased in winter pea cultivars because of the frost damage experienced on the apex of the main stem. Such conditions were encountered during the first months of the growing cycle (December–February; Fig. 1) which corresponds to the emergence of the lateral branches (Fig. 5B).

Flowering is a crucial stage of the growing cycle that has been widely studied and used in order to model pea growth. Truong and Duthion (1993) showed that the time of flowering is a function of leaf appearance rate and position of the node bearing the first flower. The reproductive development of pea cultivars was therefore characterized by two main indicators: the nodal position of the first flower and its emergence time. As also reported by Jeuffroy and Sebillotte (1997), we found similar time of flowering between main stems and basal branches (although these were produced later) for all cultivars and cropping systems. Furthermore, the position of the first flowering node was similar among the genotypes and cropping systems. Some authors also showed that for a given genotype, the position of the first flowering node was constant over various conditions (Roche *et al.* 1998; Munier-Jolain *et al.* 2005*b*).

Finally these results highlight that in the present experiment, the morphogenesis of pea was mainly determined by the genotype and was only little affected by the competition with wheat. This suggests that the architectures of pea and wheat may be quite similar, so that the environmental conditions perceived by plants in the canopy (phylloclimate; Chelle 2005) were not strongly different between sole pea crops and wheat–pea mixtures. Functional–structural models (Vos *et al.* 2010; DeJong *et al.* 2011) are able to take into account the explicit architecture of plants and its interactions with physiological processes and environmental conditions. Such models therefore constitute suitable tools for assessing these hypotheses and can in particular be used to characterize the microclimate perceived by plants located in mono- and multi-specific stands.

## Conclusion

To our knowledge, the present study is the first to compare the morphogenesis of pea grown in sole stands with that of pea grown intercropped with wheat. On the one hand, the present results show that most of the assessed parameters of pea morphogenesis (phenology, branching, final number of phytomers and their kinetics of appearance) were mainly dependent on the considered genotype. This emphasizes the importance of the selection of cultivars, in particular for intercropping systems, as this will determine the level of competition and complementarity between the component species. On the other hand, there was a low variability of pea morphogenesis between sole and mixed stands except for plant height and branching of the late cultivar 886/01. Complementary studies on wheat–pea mixtures under contrasting levels of nitrogen fertilization are now needed to provide information on how nitrogen would affect plant morphogenesis and inter-specific competition. The information provided in the present study can be used for modelling pea morphogenesis in pure and mixed stands and therefore contributes to a better understanding of the functioning of cereal–legume IC. This kind of approach is also well suited for the identification of plant traits to be integrated in the definition of plant ideotypes.

## Sources of Funding

This research was supported by ‘La Région Pays de la Loire’, France through a Ph.D. fellowship to R.B. The research of D.C. and A.E.-G. was partially funded by ‘La Région Poitou-Charentes’, France.

## Contributions by the Authors

All authors have contributed substantially to this manuscript. R.B. completed the writing and was involved in each step of the experimentation and analysis. D.C. and A.E.G. were actively involved in the conception and design of the experiment as well as in the analyses and writing of the manuscript. S.P. was involved in the conception of the experiment and also performed the measurements. P.H. was involved in database programming and data analysis.

## Conflicts of Interest Statement

None declared.
